# Factors associated with birth preparedness and complication readiness knowledge among pregnant women before implementation of a culturally adapted training intervention in rural Iringa, Tanzania

**DOI:** 10.3389/fgwh.2026.1883736

**Published:** 2026-08-14

**Authors:** Rosalia Mwenda, Saada Seif, Rehema Stephano, Fabiola Moshi

**Affiliations:** 1 Department of Nursing Management and Education, The University of Dodoma, Dodoma, Tanzania; 2 Department of Public Health, The University of Dodoma, Dodoma, Tanzania; 3 Department of Kiswahili, The University of Dodoma, Dodoma, Tanzania; 4 Department of Clinical Nursing, The University of Dodoma, Dodoma, Tanzania

**Keywords:** antenatal care, birth preparedness and complication readiness, danger signs, knowledge, maternal health, pregnancy, rural Tanzania

## Abstract

**Background:**

Birth preparedness and complication readiness (BPCR) is an important strategy for reducing delays in recognizing obstetric complications and promoting timely use of maternal health services. However, evidence on factors associated with BPCR knowledge among pregnant women in rural Tanzania remains limited. This study aimed to identify factors associated with BPCR knowledge among pregnant women before implementation of a culturally adapted training intervention in rural Iringa, Tanzania.

**Methods:**

A quantitative analytical cross-sectional study was conducted among pregnant women attending public antenatal care clinics in rural Iringa. Participants were selected using a multistage sampling procedure followed by systematic sampling from antenatal care registers. Data were collected using a structured interviewer-administered questionnaire assessing BPCR knowledge across six maternal and newborn health domains. Bivariate analyses were performed using the Mann–Whitney U and Kruskal–Wallis tests, while multivariable linear regression with heteroscedasticity-consistent standard errors was used to identify factors independently associated with BPCR knowledge.

**Results:**

A total of 176 pregnant women participated. The mean age was 26.47 ± 6.13 years, and most participants were married (86.9%), multigravida (82.4%), and had primary education (68.8%). The mean BPCR knowledge score was 21.59 ± 8.38 out of 62 (34.8% of the maximum score), indicating suboptimal knowledge. Knowledge was highest for antenatal care (76.9%) and lowest for postpartum (20.4%), newborn (25.4%), and labor-related danger signs (28.6%). In bivariate analyses, age, marital status, living with a partner, and gravidity were significantly associated with BPCR knowledge. After adjustment, only maternal age below 20 years remained independently associated with lower knowledge scores (*β* = −6.15, 95% CI: −9.59 to −2.72; *p* < 0.001). The model explained 11.3% of the variability in BPCR knowledge (adjusted R^2^ = 7.0%).

**Conclusion:**

BPCR knowledge among pregnant women attending antenatal care in rural Iringa was suboptimal, particularly regarding the recognition of obstetric and neonatal danger signs. Pregnant women aged under 20 years had significantly lower BPCR knowledge and should be prioritized for targeted antenatal education. Strengthening culturally appropriate BPCR education within routine antenatal care may help address knowledge gaps among pregnant women in rural Tanzania.

## Background

Maternal mortality remains a major public health concern worldwide, particularly in low- and lower-middle-income countries, where approximately 260,000 women die annually from preventable complications related to pregnancy, childbirth, and the postpartum period, equivalent to nearly 700 deaths each day (1). The leading causes of maternal mortality include postpartum hemorrhage, hypertensive disorders of pregnancy, and pregnancy-related infections (1, 2). Least developed countries account for nearly 60% of global maternal deaths, with an estimated maternal mortality ratio of 313 deaths per 100,000 live births (1).

Sub-Saharan Africa continues to bear a disproportionate burden of global maternal mortality, particularly among low- and lower-middle-income countries, with an estimated maternal mortality ratio of 454 deaths per 100,000 live births, accounting for approximately 70% of global maternal deaths (1). This figure remains substantially higher than the Sustainable Development Goal 3 target of reducing maternal mortality to fewer than 70 deaths per 100,000 live births by 2030 (2). Several factors contribute to the persistently high maternal mortality burden, including inadequate access to quality maternal healthcare services, delayed care-seeking, and insufficient birth preparedness and complication readiness among pregnant women and their families (1, 3, 4).

The Birth Preparedness and Complication Readiness (BPCR) strategy is strongly recommended by the World Health Organization to improve timely access to skilled maternal healthcare and reduce maternal and neonatal mortality (5, 6). BPCR emphasizes the importance of pregnant women and their families preparing in advance for safe childbirth and potential obstetric emergencies (7). The strategy includes identifying a skilled birth attendant, selecting a health facility for delivery, saving money, arranging transportation for childbirth, and recognizing danger signs during pregnancy, childbirth, and the postpartum period (8). Adequate knowledge of BPCR has been associated with increased utilization of skilled birth services, improved care-seeking behavior, and better maternal health outcomes (9, 10).

However, despite the recognized importance of BPCR, knowledge levels remain suboptimal in rural settings across many countries, particularly in Sub-Saharan Africa (11–13). Evidence from limited-resource settings suggests that BPCR knowledge is influenced by several social, demographic, and structural factors, including maternal education, occupation, parity, access to health information, and socioeconomic status (14, 15). These factors are important because they may indicate differences in women's access to maternal health information, decision-making capacity, and ability to prepare for childbirth and potential complications. Women with limited educational attainment have been reported to have lower levels of awareness of maternal health information and BPCR-related concepts, whereas women with higher educational attainment may have greater access to and ability to interpret health information (16). Identifying groups with lower BPCR knowledge can help health programs develop targeted education strategies rather than applying uniform approaches to all pregnant women.

In Tanzania, maternal mortality remains a significant public health concern, particularly in rural settings such as the Iringa Region, where women may experience challenges related to access to maternal health information, healthcare services, and timely care-seeking. The maternal mortality ratio in Tanzania remains high at approximately 104 deaths per 100,000 live births (17, 18). Although maternal deaths result primarily from direct obstetric complications, including postpartum hemorrhage, hypertensive disorders, and pregnancy-related infections, their occurrence is influenced by broader factors such as delayed access to quality maternal healthcare, limited maternal health information, and health system challenges (17, 18). Rural areas, including the Iringa Region, face additional challenges such as inadequate health education during antenatal care (ANC) and sociocultural factors that may influence maternal health-seeking behaviors (19, 20). The region also represents a culturally distinct setting where local beliefs, communication patterns, and social contexts may influence how women access and interpret maternal health information.

Although several studies in Tanzania have examined BPCR knowledge among pregnant women, evidence regarding factors associated with BPCR knowledge remains inconsistent across settings (21–23). Previous studies have reported associations with maternal education, occupation, parity, socioeconomic status, and access to health information; however, these relationships have not been consistently observed across different populations and contexts (22, 23). Moreover, much of the existing evidence has focused on describing BPCR knowledge levels and practices rather than identifying factors independently associated with variations in knowledge (21, 23). Evidence from rural and culturally distinct settings, including the Iringa Region, remains limited. Iringa Region has a largely rural population and includes culturally distinct communities whose beliefs, communication patterns, and interpretations related to pregnancy and childbirth may influence access to, understanding of, and responses to maternal health information (24). Therefore, examining BPCR knowledge within specific cultural contexts is important for developing appropriate maternal health education strategies.

Although ANC provides an important platform for delivering BPCR education, the effectiveness of health messages may depend on whether they are understood and perceived as meaningful within the sociocultural context of the target population (25). The Iringa Region, characterized by predominantly rural communities and culturally distinct populations, provides an important context for examining factors influencing maternal health knowledge and communication (26). The Hehe community in Iringa represents such a culturally distinct population, where social and cultural factors may influence the interpretation of and response to maternal health information and preparedness practices (24). Examining sociodemographic characteristics as potential factors associated with BPCR knowledge, rather than merely describing participant profiles, is important for identifying disparities in maternal health information access and informing targeted ANC education strategies.

However, it remains unclear which maternal characteristics are independently associated with BPCR knowledge among pregnant women in rural and culturally distinct settings in Tanzania, particularly before implementation of context-specific maternal health education interventions. Therefore, this study aimed to identify factors associated with BPCR knowledge among pregnant women attending the ANC before the implementation of a culturally adapted training intervention in rural Iringa, Tanzania. Identifying factors associated with lower BPCR knowledge provides evidence to support targeted antenatal education and inform the design of culturally appropriate maternal health interventions.

## Methods and materials

### Study setting

The study was conducted in Iringa Region, located in the Southern Highlands of Tanzania. The region has a largely rural population, with agriculture being the main economic activity. Maternal healthcare services are provided through a network of public health facilities, including dispensaries, health centers, and hospitals, which offer routine ANC services, health education, and referral services for women requiring specialized care (27). Despite the availability of maternal health services, sociocultural factors and barriers related to access and care-seeking continue to influence maternal health behaviors (20). The Iringa Region has a maternal mortality ratio of 105.5 deaths per 100,000 live births due to pregnancy-related causes, which remains above the Sustainable Development Goal 3 target of fewer than 70 deaths per 100,000 live births by 2030. Approximately 65% of pregnant women in the region completed four or more ANC visits (28).

### Study design

A quantitative analytical cross-sectional study was conducted to assess factors associated with BPCR knowledge among pregnant women before the implementation of a culturally adapted BPCR education intervention. This baseline analysis used data collected before the intervention from a parent quasi-experimental study that employed a pretest–posttest design to evaluate the effect of the intervention on BPCR knowledge, attitudes, and practices among pregnant women in rural Iringa, Tanzania.

## Study population and participants’ characteristics

### Inclusion criteria

The study included pregnant women from the indigenous Hehe ethnic group who were in the first or second trimester of pregnancy and attending ANC services at selected health facilities. Women in early pregnancy were included because they were expected to have sufficient time to participate in the maternal health education intervention and complete scheduled follow-up assessments. Women who were able to communicate effectively, participate in study procedures, and provide informed consent were eligible. Women attending either their first ANC visit or subsequent ANC visits were eligible if they met the study criteria. Women in the third trimester were excluded because they were outside the predefined study population. Pregnant women below 18 years were eligible according to the study protocol; however, parental/guardian consent and participant assent would have been obtained where required. No participants below 18 years were recruited during the study period.

### Exclusion criteria

Women with high-risk pregnancies, severe pregnancy-related complications, or serious pre-existing medical conditions were excluded because these conditions could affect their ability to participate safely in scheduled study activities and complete follow-up assessments. These included severe pregnancy-related complications such as intrauterine fetal death or pregnancy-induced hypertension and serious pre-existing conditions such as heart disease or stroke. Women employed within the healthcare system were excluded because their professional exposure to maternal health information could influence baseline BPCR knowledge assessment. Women participating in other programs or research studies were excluded to minimize potential interference with scheduled study activities and follow-up assessments. Women who declined informed consent were not enrolled.

### Sample size and sampling procedure

#### Sample size

This study used baseline data from pregnant women enrolled in a larger quasi-experimental study evaluating the effect of a culturally adapted BPCR training intervention in rural Iringa, Tanzania. The sample size for the parent intervention study was determined using the formula for comparing two independent means implemented in WINPEPI software, based on an expected difference in mean scores between the intervention and control groups of 5.2 and 3.4, respectively, reported in a previous study (29). Assuming a 95% confidence level and 80% statistical power, the minimum required sample size was estimated to be 164 participants. To account for a potential 10% non-response rate during recruitment, the sample size was increased to 176 participants. For the present cross-sectional analysis, all 176 pregnant women who completed the baseline assessment were included.

#### Sampling procedure

A multistage sampling approach was used to select study participants in rural districts of the Iringa Region, Tanzania. The first stage involved purposive selection of eligible rural districts, followed by random selection of divisions, wards, and villages, and systematic random sampling of eligible pregnant women from ANC registers. According to the National Bureau of Statistics (30), the region comprises four rural districts, 31 divisions, 106 wards, and 360 villages. In the first stage, two rural districts were selected from three eligible districts using simple random sampling through a lottery method.

In the second stage, two divisions were randomly selected from each district, resulting in four divisions. In the third stage, two wards were randomly selected from each division, yielding a total of eight wards. In the fourth stage, two villages were randomly selected from each of the eight wards, resulting in 16 villages included in the study.

In the final stage, public health facilities providing ANC services within the selected villages were identified as recruitment sites. All eligible public health facilities were included in the study; no additional facility-level random selection was performed. Pregnant women attending ANC services at these facilities were then recruited using systematic random sampling from ANC registers. Before sampling, the ANC register was screened to identify eligible women belonging to the Hehe ethnic group. Ethnicity was confirmed during ANC registration records and participant verification before inclusion in the sampling frame. The sampling interval was calculated using k = N/n, where N represented the total number of eligible pregnant women registered at the selected facilities and n represented the required sample size. Every k^th^ eligible pregnant woman was selected until the required sample size was achieved.

### Conceptual framework

**Figure 1:**
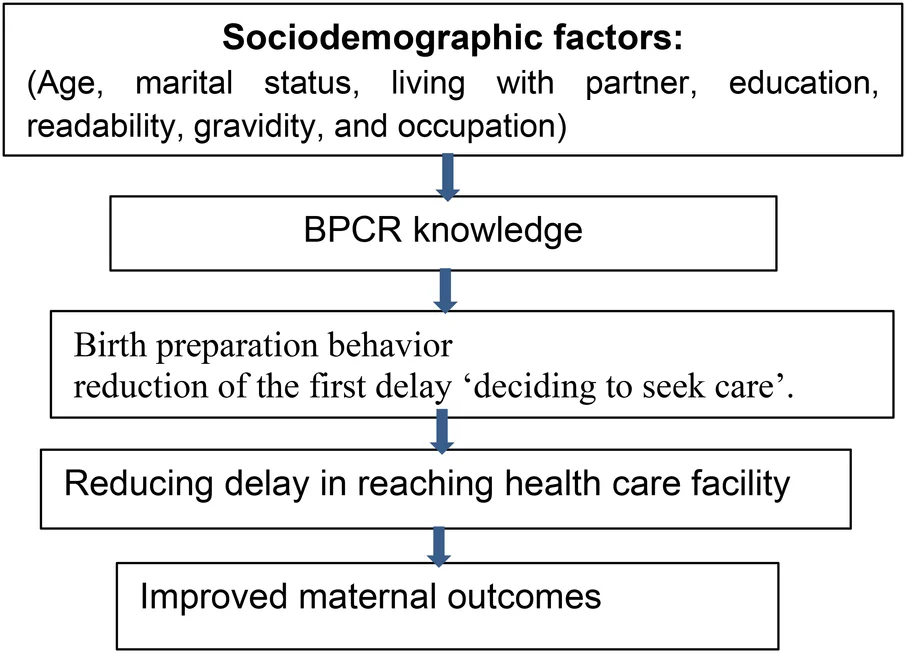
Conceptual framework model.

This study was guided by the Three Delays Model developed in the United States by Sereen Thaddeus and Deborah Maine in 1994 (31), which explains delays in accessing maternal health care. The first delay, which involves deciding to seek care, is influenced by maternal knowledge, awareness of obstetric danger signs, and sociocultural factors such as age, gravidity, marital status, education, and occupation (31). These factors may also influence BPCR knowledge, which in turn affects birth preparedness and the recognition of pregnancy complications (Figure 1).

### Study variable

The dependent variable in this study was the knowledge of BPCR among pregnant women. The independent variables included maternal age, gestational age, gravidity, religion, marital status, living with a partner, educational level, reading ability, and occupation.

### Measurement of BPCR knowledge

The outcome variable was BPCR knowledge. The questionnaire was designed to cover six BPCR-related domains: danger signs during pregnancy (14 items), labor and childbirth (8 items), the postpartum period (11 items), the newborn period and essential newborn care (14 items), birth preparedness (8 items), and ANC (7 items). The final BPCR knowledge score was calculated based on the 62 items collected. Each item had a dichotomous response and was scored as 1 for a correct response indicating knowledge and 0 for an incorrect response. The overall BPCR knowledge score was calculated by summing responses across the 62 items, giving a possible range of 0 to 62. Higher scores indicated greater BPCR knowledge. To permit comparison with studies employing instruments of different lengths, the knowledge score was also expressed as a percentage of the maximum attainable score.

Sociodemographic and obstetric characteristics were collected through face-to-face interviews using a structured questionnaire. Maternal age was recorded in completed years and gestational age in completed months of pregnancy. Gravidity was categorized as primigravida or multigravida. Marital status was categorized as single or married. Educational level was categorized as primary education or secondary education and above. Reading ability was categorized as reads easily or reads with difficulty. Occupation was categorized as peasant or businesswoman/employee. Continuous variables were recorded numerically, and categorical variables were coded according to their respective response categories.

### Data collection tool

Data was collected using a structured questionnaire adapted from the BPCR monitoring tools developed by Jhpiego in collaboration with Family Care International for safe motherhood program assessment in low- and middle-income countries (32). The questionnaire included items assessing sociodemographic characteristics, knowledge of danger signs during pregnancy, labor, and childbirth; postpartum and newborn periods; ANC and birth preparation. The tool consisted of closed-ended questions, including yes/no, multiple-response. Previous studies using similar tools reported acceptable internal consistency, with Cronbach's alpha values ranging from 0.72 to 0.85 (33–35)**.**

### Validity and reliability of the tool

The tool was adapted rather than adopted unchanged. Adaptation involved expert review, translation into Kiswahili with back-translation, and pretesting among pregnant women from an area outside the study setting. Previous use of the tool in Tanzania and other low- and middle-income countries supported its content relevance for assessing BPCR knowledge. Internal consistency was assessed in the present study using the Kuder–Richardson Formula 20 (KR-20). The overall knowledge scale demonstrated high internal consistency (KR-20 = 0.892), supporting the use of the total knowledge score as the primary outcome measure. Domain-level coefficients ranged from 0.414 to 0.675, with lower values observed in shorter domains. The total knowledge score was used as the outcome variable in the analysis.

### Data collection method and procedures

Data was collected using a researcher-administered structured questionnaire through face-to-face interviews conducted in a quiet and private space at ANC clinics. Interviews were conducted by trained research assistants who were qualified midwives fluent in both Swahili and Hehe and working in health facilities outside the study area. The research assistants had professional experience ranging from 10 to 18 years and received orientation on the study objectives, data collection procedures, and ethical considerations prior to data collection. The principal investigator supervised the overall data collection process, including training and supervising research assistants on study procedures and ethical considerations. Prior to data collection, participants were informed about the purpose of the study, and written informed consent was obtained. Each interview lasted approximately 20–25 minutes, and clarifications were provided where necessary to ensure participants understood the questions before responding.

### Data management and statistical analysis

Data were analyzed using IBM SPSS Statistics version 27, with verification in Python (pandas, SciPy and stats models). The dataset was checked for completeness, and no missing data were identified; all eligible participants were therefore included. Prior to inferential analysis, the distribution of the BPCR knowledge score was examined using the Shapiro–Wilk test. The score departed significantly from normality (W = 0.973, *p* = 0.002), and non-parametric methods were therefore adopted as the primary approach for bivariate comparison. Parametric equivalents were computed in parallel as a sensitivity check and did not alter any substantive conclusion. Statistical significance was assessed at *p* < 0.05 throughout.

Descriptive statistics were used to summarize participants’ sociodemographic characteristics and study variables. Continuous variables were presented as means and standard deviations, while categorical variables were summarized using frequencies and percentages.

Bivariate analysis was performed to assess the unadjusted association between each sociodemographic characteristic and the BPCR knowledge score. The Mann–Whitney U test was used for characteristics with two levels and the Kruskal–Wallis test for characteristics with three or more levels, both being appropriate to the non-normal distribution of the outcome. Subsequently, all measured sociodemographic variables were entered simultaneously into a multivariable linear regression model to identify factors independently associated with BPCR knowledge while controlling for the remaining variables. Living with a partner was not entered into the multivariable model because it was almost perfectly collinear with marital status, and entering both produced an unstable solution; marital status was retained. Variance inflation factors were inspected in the fitted model. Heteroscedasticity-consistent (HC3) standard errors were used because the model residuals were not normally distributed. Regression coefficients (*β*) and 95% confidence intervals are reported, with statistical significance assessed at *p* < 0.05.

### Ethics approval, consent, and confidentiality

Before data collection, ethical clearance and a permission letter were obtained from the Research Review Committee Board in the Research and Publication Office of the University of Dodoma (number MA.84/261/86/11). A written informed consent was obtained from all participants before their inclusion in the study. All procedures performed in this study involving human participants were conducted in accordance with institutional ethical standards and principles of the Declaration of Helsinki. Permission to conduct the study in the Iringa region was obtained from the Permanent Secretary, President's Office, Regional Administration, and local government reference number AB.307/323/01"R”/70. Permission to use the ANC guidelines for the study was requested from the Ministry of Health, reference number PA.104/282/02. The permission to collect data in the Iringa region was obtained from the Iringa Regional Secretary, reference number Na.FA.225/265/01/J/281. The permission to collect data in the Mufindi and Kilolo districts was obtained from district directors; in the Mufindi district, the permission reference number was Na. HW/MUF/S.40/43/VOL.IV/39, and the Kilolo reference number was Na. KDC/S.10/4/VOLL.VIII/193. During the study, confidentiality was upheld; participants assigned numbers rather than using their names. Cultural respect and sensitivity were prioritized throughout the study to ensure that the training content and implementation do not conflict with the participants’ beliefs or practices.

## Results

### Sociodemographic characteristics of pregnant women

Table 1 presents the sociodemographic characteristics of the 176 pregnant women enrolled in the study. The mean age of participants was 26.47 ± 6.13 years (range 18–44 years), and most were aged 20–34 years (71.0%). The mean gestational age was 4.66 ± 1.21 months, and most participants were beyond the first trimester (82.4%). The majority had experienced two or more pregnancies (82.4%), were married (86.9%), and were living with their partners (84.1%). Most had attained primary education (68.8%), reported being able to read easily (95.5%), and were peasants (89.8%) (Table 1).

**Table 1:** Sociodemographic characteristics of pregnant women (*n* = 176).

Variable	Category	*n* (%)/Mean ± SD
Age (years)	Mean ± SD (Min–Max)	26.47 ± 6.13 (18–44)
	< 20	28 (15.9)
	20–34	125 (71.0)
	≥ 35	23 (13.1)
Gestational age	Mean ± SD (Min–Max)	4.66 ± 1.21 (2–6)
	≤ 3 months	31 (17.6)
	4–6 months	145 (82.4)
Gravidity	Primigravida	31 (17.6)
	Multigravida	145 (82.4)
Marital status	Single	23 (13.1)
	Married	153 (86.9)
Living with a partner	No	28 (15.9)
	Yes	148 (84.1)
Educational level	Primary education	121 (68.8)
	Secondary education and above	55 (31.2)
Reading ability	Easily	168 (95.5)
	With difficulty	8 (4.5)
Occupation	Peasant	158 (89.8)
	Business woman/employed	18 (10.2)

SD, standard deviation.

### BPCR knowledge scores among pregnant women

The BPCR knowledge score among the 176 pregnant women ranged from 5 to 57 out of a possible 62, with a mean of 21.59 ± 8.38. This corresponds to 34.8% of the maximum attainable score, indicating that on average participants correctly identified about one third of the BPCR knowledge items. Knowledge was not uniform across domains. It was highest for antenatal care (76.9% of the domain maximum) and birth preparedness (47.6%) and lowest for the recognition of danger signs, reaching only 30.8% for pregnancy, 28.6% for labor and childbirth, 25.4% for the newborn period, and 20.4% for the postpartum period. The wide standard deviation indicates substantial variability in knowledge among respondents (Table 2).

**Table 2:** BPCR knowledge scores among pregnant women (*N* = 176).

Domain (number of items)	Mean ± SD	% of maximum
Danger signs in pregnancy (14)	4.31 ± 2.31	30.8
Danger signs in labor and childbirth (8)	2.28 ± 1.67	28.6
Danger signs in the postpartum period (11)	2.24 ± 1.87	20.4
Newborn danger signs and essential care (14)	3.56 ± 2.15	25.4
Birth preparedness (8)	3.81 ± 1.51	47.6
Antenatal care (7)	5.39 ± 1.11	76.9
Overall BPCR knowledge score (62)	21.59 ± 8.38 (5–57)	34.8

SD, standard deviation. The BPCR knowledge scale comprised 62 items, with total scores ranging from 0 to 62.

### Bivariate association between sociodemographic characteristics and BPCR knowledge score

Table 3 shows the association between sociodemographic characteristics and BPCR knowledge scores. Because the knowledge score departed significantly from a normal distribution (Shapiro–Wilk W = 0.973, *p* = 0.002), the Mann–Whitney U test was used for two-level characteristics and the Kruskal–Wallis test for age, which had three levels. Four characteristics were significantly associated with BPCR knowledge. Knowledge differed significantly across age groups (H = 13.51, *p* = 0.001): women aged under 20 years scored markedly lower (mean 16.04) than those aged 20–34 years (22.70) or 35 years and above (22.30). Married women scored higher than single women (median 22 versus 18; U = 1170.0, *p* = 0.010), women living with a partner scored higher than those who were not (median 22 versus 19; U = 1305.0, *p* = 0.002), and multigravida women scored higher than primigravid women (median 22 versus 20; U = 1723.5, *p* = 0.042).

**Table 3:** Bivariate association between sociodemographic characteristics and BPCR knowledge scores among pregnant women (*N* = 176).

Variable	Category	n	Mean ± SD	Median (IQR)	Test statistic	p
Age (years)					H = 13.51	0.001*
	< 20	28	16.04 ± 7.43	17 (9–22)		
	20–34	125	22.70 ± 7.60	22 (19–28)		
	≥ 35	23	22.30 ± 10.86	22 (14–26)		
Gestational age					U = 2276.5	0.912
	≤ 3 months	31	21.61 ± 7.86	23 (18–24)		
	4–6 months	145	21.59 ± 8.51	21 (16–26)		
Gravidity					U = 1723.5	0.042*
	Primigravida	31	18.68 ± 7.31	20 (13–24)		
	Multigravida	145	22.21 ± 8.48	22 (16–28)		
Marital status					U = 1170.0	0.010*
	Single	23	17.09 ± 7.99	18 (10–24)		
	Married	153	22.27 ± 8.25	22 (18–27)		
Living with a partner					U = 1305.0	0.002*
	No	28	17.07 ± 7.54	19 (10–22)		
	Yes	148	22.45 ± 8.28	22 (18–27)		
Educational level					U = 3140.0	0.550
	Primary education	121	21.14 ± 8.21	21 (16–26)		
	Secondary and above	55	22.58 ± 8.73	22 (18–26)		
Reading ability					U = 675.0	0.986
	Easily	168	21.59 ± 8.38	22 (16–26)		
	With difficulty	8	21.62 ± 8.94	20 (16–30)		
Occupation					U = 1614.0	0.349
	Peasant	158	21.77 ± 8.45	22 (16–26)		
	Business woman/employed	18	20.00 ± 7.72	20 (15–24)		

Data are presented as mean ± SD and median (IQR). H = Kruskal–Wallis test statistic; U = Mann–Whitney U test statistic. Non-parametric tests were used because BPCR knowledge scores were not normally distributed. *n* = number of participants; SD = standard deviation; IQR = interquartile range. *p* < 0.05 was considered statistically significant (indicated by *).

No statistically significant associations were found between BPCR knowledge scores and gestational age (U = 2276.5, *p* = 0.912), educational level (U = 3140.0, *p* = 0.550), reading ability (U = 675.0, *p* = 0.986), or occupation (U = 1614.0, *p* = 0.349) (Table 3).

### Multivariable linear regression analysis of factors associated with BPCR knowledge scores among pregnant women

Table 4 presents the multivariable linear regression analysis of factors associated with BPCR knowledge. All sociodemographic characteristics were entered simultaneously. Age was modelled in three categories (under 20, 20–34, and 35 years and above), with 20–34 years as the reference, because the relationship between age and knowledge was not linear: knowledge was low among women under 20 but comparable in the two older groups. Educational level, reading ability and occupation were each entered as two categories, and living with a partner was excluded because it is almost perfectly collinear with marital status. Heteroscedasticity-consistent (HC3) standard errors were used because the residuals were not normally distributed.

**Table 4:** Multivariable linear regression analysis of factors associated with BPCR knowledge scores among pregnant women (*N* = 176).

Variable	*β*	SE	t	p	95% CI
*Age (years)*					
Under 20	−6.15	1.75	−3.51	< 0.001*	−9.59 to −2.72
35 and above	−0.80	2.47	−0.32	0.746	−5.64 to 4.04
20–34 (Ref)	–	–	–	–	–
*Gestational age (months)*	−0.20	0.50	−0.40	0.687	−1.19 to 0.78
*Gravidity*					
Primigravida	0.24	1.64	0.14	0.885	−2.97 to 3.44
Multigravida (Ref)	–	–	–	–	–
*Marital status*					
Single	−2.94	2.36	−1.25	0.213	−7.56 to 1.68
Married (Ref)	–	–	–	–	–
*Educational level*					
Secondary and above	1.48	1.47	1.01	0.313	−1.40 to 4.37
Primary (Ref)	–	–	–	–	–
*Reading ability*					
With difficulty	−0.15	3.02	−0.05	0.959	−6.07 to 5.76
Reads easily (Ref)	–	–	–	–	–
*Occupation*					
Business woman/employed	−2.60	2.08	−1.25	0.211	−6.68 to 1.47
Peasant (Ref)	–	–	–	–	–
*Model summary*	R^2^ = 0.113; adjusted R^2^ = 0.070; F (8, 167) = 2.65; *p* = 0.009				

β, unstandardized regression coefficient; SE, standard error; CI, confidence interval; Ref, reference category. HC3 heteroscedasticity-consistent standard errors were used. Reference categories: age 20–34 years, multigravida, married, primary education, reads easily, and peasant. Living with a partner was excluded because of collinearity with marital status.

*p < 0.05.

After mutual adjustment, maternal age under 20 years was independently associated with lower BPCR knowledge: women aged under 20 scored on average 6.15 points lower than those aged 20–34 years (*β* = −6.15, 95% CI: −9.59 to −2.72, *p* < 0.001). No other characteristic was independently associated with knowledge. Being 35 years or older (*β* = −0.80, *p* = 0.746), single marital status (*β* = −2.94, *p* = 0.213), secondary or higher education (*β* = 1.48, *p* = 0.313), occupation (*β* = −2.60, *p* = 0.211), gravidity (*β* = 0.24, *p* = 0.885), reading ability (*β* = −0.15, *p* = 0.959) and gestational age (*β* = −0.20, *p* = 0.687) were all non-significant.

The overall model was statistically significant and explained approximately 11% of the variation in BPCR knowledge [R^2^ = 0.113, adjusted R^2^ = 0.070; F(8, 167) = 2.65, *p* = 0.009]. All variance inflation factors were below 1.6, indicating no problematic collinearity.

The bivariate associations observed for marital status and gravidity were attenuated to non-significance after adjustment, indicating that they were largely explained by their shared association with maternal age and with one another. Maternal age under 20 years was the single characteristic that remained independently associated with BPCR knowledge.

This identifies adolescent and very young pregnant women as a specific subgroup with markedly lower BPCR knowledge, toward whom antenatal health education may be most usefully directed (Table 4).

## Discussion

The present study examined BPCR knowledge and its sociodemographic correlates among pregnant women attending public antenatal care clinics in rural Iringa, Tanzania. Three principal findings emerged. First, overall BPCR knowledge was low, averaging only one third of the maximum attainable score. Second, knowledge was particularly poor for obstetric and neonatal danger signs despite universal ANC attendance. Third, after multivariable adjustment, maternal age under 20 years was the only sociodemographic characteristic independently associated with BPCR knowledge, whereas education, gravidity, marital status, and occupation were not independently associated. The sociodemographic profile of the participants provides context for interpreting these findings.

BPCR knowledge in this study averaged approximately one third of the maximum attainable score, despite universal ANC attendance and a predominantly multigravida sample. These findings suggest that ANC attendance alone may not be sufficient to ensure adequate BPCR knowledge. The domain profile makes the point more sharply. Knowledge of ANC, which largely records whether a woman attended clinic and was counselled, reached 76.9% of its maximum, whereas recognition of danger signs, which a woman must exercise unaided and often at night and far from a facility, reached only 20.4% in the postpartum period and 25.4% in the newborn period. These findings indicate that although knowledge related to ANC was relatively high, recognition of obstetric and neonatal danger signs remained limited across pregnancy, childbirth, the postpartum period, and the newborn period. Similar patterns of low recognition of obstetric and neonatal danger signs have been reported in other Tanzanian settings and across sub-Saharan Africa, suggesting that routine ANC contacts should place greater emphasis on danger-sign recognition alongside general birth preparedness education (34, 36).

Maternal age under 20 years was the only sociodemographic characteristic independently associated with lower BPCR knowledge after multivariable adjustment. This finding is consistent with evidence that adolescent pregnancy is often associated with limited reproductive health knowledge, reduced decision-making autonomy, and restricted access to maternal health information (37–39). In rural Tanzania and similar low-resource settings, these factors may be compounded by greater reliance on partners or family members for health-related decision-making and fewer opportunities to access accurate maternal health information (40–42). Within the Three Delays Model (31), these findings suggest that adolescent and very young pregnant women may be particularly vulnerable to the first delay in recognizing complications and deciding to seek appropriate care. Although care-seeking behavior was not assessed directly, the findings identify younger pregnant women as a priority group for targeted BPCR education during routine antenatal care.

Apart from maternal age, none of the sociodemographic characteristics examined independently predicted BPCR knowledge after multivariable adjustment. Although multigravida women, married women, women living with partners, and those with higher educational attainment demonstrated higher BPCR knowledge in unadjusted analyses, these associations disappeared after controlling for potential confounders. This suggests that the apparent advantages associated with previous pregnancy experience, partnership, or educational attainment were largely explained by their interrelationships with age and reproductive experience rather than representing independent determinants of BPCR knowledge. The absence of significant associations for education and occupation may also reflect the relative homogeneity of the study population, in which most participants had only primary education and were engaged in subsistence farming, limiting the variability required to detect meaningful differences. These findings differ from studies conducted in Tanzania, Ethiopia, Egypt, and Nigeria, where higher educational attainment and socioeconomic status were consistently associated with better BPCR knowledge and utilization (11, 40, 43, 44). The discrepancy may indicate that, within this rural population, BPCR knowledge is influenced less by sociodemographic characteristics than by contextual factors not measured in this study, including the quality and consistency of antenatal counselling, household economic resources, access to maternal health information, and geographical accessibility of health facilities. This interpretation is bolstered by the regression model, which revealed that the assessed sociodemographic variables accounted for less than 7% of the variation in BPCR knowledge. Consistent with the Three Delays Model, improving BPCR knowledge in this setting is therefore likely to depend more on strengthening the content and quality of antenatal education than on demographic characteristics alone.

Perhaps the most important finding was that the sociodemographic characteristics included in the multivariable model accounted for only a modest proportion of the variability in BPCR knowledge (R^2^ = 11.3%; adjusted R^2^ = 7.0%), indicating that demographic characteristics alone may be insufficient to explain differences in BPCR knowledge. This suggests that BPCR knowledge among women attending rural public ANC clinics in Iringa may be influenced predominantly by factors beyond the demographic characteristics measured in this study. Unmeasured contextual factors, including the quality and consistency of antenatal counseling, household socioeconomic conditions, previous exposure to maternal health information, cultural beliefs, and geographical accessibility of health services, may account for much of the remaining variation in BPCR knowledge.

### Theoretical implications

The findings are consistent with the Three Delays Model, which proposes that maternal deaths may result from delays in recognizing complications and deciding to seek appropriate care at the household level before reaching a health facility (31). The domain-specific pattern of knowledge observed in this study suggests that the principal knowledge deficit lies within the first delay. Although women demonstrated relatively better knowledge of birth preparedness and ANC, their recognition of obstetric and neonatal danger signs was substantially lower, potentially limiting timely recognition of complications and appropriate care-seeking.

The findings also align with the Health Belief Model, particularly the constructs of perceived susceptibility and perceived severity, which suggest that individuals’ health actions are influenced by their perceived risk of experiencing a health problem and the seriousness attributed to that problem (45, 46). In this context, limited recognition of danger signs may constrain women's ability to perceive pregnancy complications as potential threats requiring urgent care. Although these constructs were not measured directly in this study, improving women's knowledge of danger signs may enhance perceived risk and support timely care-seeking behaviors. Future studies should examine these theoretical constructs directly to better understand behavioral pathways through which BPCR education influences maternal health practices. Notably, the sociodemographic characteristics examined explained only a modest proportion of the variation in BPCR knowledge, suggesting that other contextual factors, including the quality and consistency of antenatal education, access to maternal health information, and broader sociocultural influences, may contribute more substantially to knowledge differences in this setting.

### Practical and policy implications

The results demonstrated the need to strengthen BPCR education within routine ANC. Because maternal age under 20 years was the characteristic most strongly and independently associated with lower BPCR knowledge, BPCR education strategies should give particular attention to adolescent and very young pregnant women, although the low overall level of knowledge indicates a need for improved BPCR education across the entire population. Routine ANC counselling should place greater emphasis on the recognition of obstetric and neonatal danger signs, which represented the weakest knowledge domain, and should be delivered using culturally appropriate messages that are responsive to local beliefs, language, and practices. Educational materials should be presented in simple formats that accommodate varying literacy levels. Where feasible, mobile health (mHealth) strategies, such as SMS reminders or mobile phone–based educational messages, could complement routine ANC counselling by reinforcing BPCR information between clinic visits, particularly among younger pregnant women. Community health workers should be engaged to reinforce BPCR messages at household and community levels, while greater involvement of male partners may strengthen household preparedness and timely decision-making.

### Strengths of the study

This study has several strengths. First, it was conducted in a rural setting where evidence on BPCR knowledge remains limited, contributing context-specific data for Tanzania. Second, participants were recruited from routine public ANC clinics, providing findings that are directly applicable to women attending public ANC services in rural Iringa, although not necessarily to pregnant women who do not attend ANC or seek care in private facilities. Third, the instrument covered six BPCR domains, and its internal consistency was verified in the present sample rather than assumed from previous applications, with the overall knowledge scale achieving strong reliability. Fourth, knowledge was analyzed as a continuous score, preserving the information that dichotomization would discard, and all sociodemographic variables were entered simultaneously into the multivariable model so that the reported estimates are mutually adjusted. Finally, the analysis reports the domain structure of knowledge rather than the total alone, which reveals a pattern of deficit that an aggregate score would have concealed.

### Limitations of the study

Despite its strengths, this study has several limitations. First, the cross-sectional analysis of baseline data precludes causal inference. Second, the facility-based sampling strategy may have introduced selection bias because participants were recruited exclusively from public ANC clinics in rural Iringa. Consequently, pregnant women who did not attend ANC, sought care at private health facilities, or lived in remote communities with limited access to public ANC services were not represented. Therefore, the findings should be generalized primarily to pregnant women attending public ANC clinics in rural Iringa and should not be generalized to all pregnant women in the region, including those who do not attend ANC, seek care at private health facilities, or live-in remote communities with limited access to public ANC services. In addition, because all participants were recruited from ANC clinics, the relationship between ANC attendance and BPCR knowledge could not be examined. Third, although data were collected by trained research assistants using a standardized interviewer-administered questionnaire, interviewer bias, recall bias, and social desirability bias cannot be excluded. Fourth, two knowledge domains, birth preparedness and ANC, showed weak internal consistency, and scores derived from them carry greater measurement error. Finally, residual confounding by unmeasured factors is likely. Household socioeconomic status, distance to health facilities, pregnancy intention, previous obstetric complications, health insurance status, ANC attendance frequency, quality of ANC counselling, and exposure to maternal health information were not measured. The relatively low explanatory power of the regression model further suggests that important determinants of BPCR knowledge remain unidentified. Future studies should incorporate these factors to develop a more comprehensive understanding of the determinants of BPCR knowledge and to inform interventions that address the informational needs of pregnant women in rural Tanzania.

## Conclusion

This baseline study found that BPCR knowledge among pregnant women in rural Iringa was low, averaging 21.59 of a possible 62 points, or approximately one third of the maximum attainable score, and that knowledge of obstetric and neonatal danger signs was substantially weaker than knowledge of ANC and general birth preparedness. Age, marital status and gravidity were each associated with knowledge in unadjusted analyses, but after mutual adjustment only maternal age under 20 years remained independently associated with lower knowledge. This identifies adolescent and very young pregnant women as a subgroup with a particular knowledge deficit, while the low overall level of knowledge indicates a broader need across the population. These findings in the importance of strengthening ANC health education for all pregnant women, with particular attention to younger women and to the recognition of danger signs. Future intervention studies should evaluate culturally tailored BPCR education programmes in rural Tanzania.

## Data Availability

The original contributions presented in the study are included in the article/Supplementary Material, further inquiries can be directed to the corresponding authors.
